# Work ability of Chinese migrant workers: the influence of migration characteristics

**DOI:** 10.1186/1471-2458-14-353

**Published:** 2014-04-13

**Authors:** Lu Han, Leiyu Shi, Liming Lu, Li Ling

**Affiliations:** 1 Faculty of Medical Statistics and Epidemiology, School of Public Health, Sun Yat-sen University, Guangzhou 510405, China; 2 Sun Yat-sen Center for Migrant Health Policy, Sun Yat-sen University, Guangzhou 510405, China; 3 Department of Health Policy and Management, Bloomberg School of Public Health, Johns Hopkins University, Baltimore, MD 21205, USA

**Keywords:** Chinese migrant workers, Work ability, Occupational health and safety

## Abstract

**Background:**

Migrant workers have become a vital labor supply to China’s economy. Their migration process and work conditions may influence their health and work ability. The work ability of migrant workers in China and the influence of the migration process on work ability have not been explored extensively in previous studies. The objective of this study is to evaluate the association of migration characteristics and work-related factors with work ability among migrant workers in the Pearl River Delta.

**Methods:**

In this cross-sectional survey, the study population consisted of 907 migrant workers from ten factories in the Pearl River Delta who were exposed to organic solvents during work. The primary dependent variable of the study was work ability, measured by the Work Ability Index (WAI). The independent variables were individual characteristics, migration characteristics, and work-related factors. Logistic regression models were used to determine the influence of different factors on work ability and three dimensions of WAI.

**Results:**

The result shows that among migration characteristics, social support was significantly associated with all three dimensions of the work ability index. Permanent migration intention and longer length of migration were negatively associated with the mental resource dimension of WAI. WAI was also influenced by individual and work-related factors.

**Conclusions:**

The findings of this study suggest that expanding migrants’ social networks and social support systems in their work place or living community, (i.e. expanding the functions of labor unions) would be an effective way to improve migrant workers’ work ability. Improving of migrant workers’ physical and psychosocial related work environments would also increase their work ability.

## Background

Over the past three decades, China’s economic development has produced the largest human migration in history, leading to a rise in urban population from 191 million in 1980, to 691 million in 2011 [1]—an increase driven largely by rural-to-urban migrant workers, those who migrate from rural areas of their original hukou (household registration) to urban areas for jobs or better lives. By 2012, the number of migrant workers had reached 263 million (19.4% of the total population) [2] and it is expected to continue increasing as China continues to urbanize. In economically developed areas such as Pearl River Delta in Guangdong Province, nearly 90% of employees in small and medium-sized enterprises are migrant workers.

Although migrant workers have become a vital labor supply to China’s economy, this population lack protection, both in health and work ability. Most migrant workers are exposed to poor working conditions and occupational hazards [2] and work long hours, up to 10–12 hours a day, 6–7 days a week [3]. Migrant workers comprise 80% of all occupational health patients, and have become the group in China’s labor force with the highest incidences of occupational diseases [4]. In addition to occupational condition, migration process is another important factor influencing their health [5-7]. In the process of migration, social support, level of acceptance in destination areas, length of detention, extent of isolation, family network, relationship with co-workers, interpersonal tensions and conflicts are the primary factors impacting migrants’ physical and mental health [8-10]. Any of these migration characteristics may damage the health of migrant workers—the main labor force of China’s manufacturing industry, and then influence the work ability and productivity of migrant workers. In the context of China’s aging population, it is increasingly important to protect the work ability of migrant workers.

Previous studies on the health of migrant workers in China have mostly used occupational diseases and injuries as indications of health. When migrant workers suffer with occupational diseases and injuries, they tend to return to their hometown, so that they cannot be selected as sample in studies based in urban areas. Furthermore, occupational diseases and injuries only reflect physical health, but neglect mental health and work ability.

The Work Ability Index (WAI) is an instrument used in clinical occupational health and research to assess work ability during health examinations and workplace surveys. The index is determined on the basis of the answers to a series of questions which take into consideration the demands of work, the worker’s health status and mental resources. It was first used in the early 1980s in Finland [11]. The Chinese version of WAI has been tested for validity and a series of studies ensued thereafter [12]. A number of studies have been conducted among different populations to reveal the relation between individual and work-related factors with WAI [13], but it has yet to be used among migrant workers. This relationship between migration-related factors and WAI has not been studied yet. In this study, we used WAI as a indication of migrant workers’ well-being, and evaluated the association of migration characteristics with work ability among migrant workers in China. The result could help deepen the understanding of the factors associated with work ability, as well as help to draw attention to establish policies to protect the work ability of migrant workers.

## Methods

### Study setting

Guangdong province has the largest number of rural-to-urban migrants in China, accounting for 24% of the inter-provincial population inflow, followed by Zhejiang (23.6%), Shanghai (12.7%) and Beijing (12.7%) [2]. Our study was conducted in the spring of 2012 in the Pearl River Delta Region of Guangdong province covering over 90% of the migrant population in the province including nine out of twenty-one cities (Government 2013). Using a three-stage stratified sampling method, two developed cities (Guangzhou and Foshan) and two less-developed cities (Zhaoqing and Qingyuan) in the Pearl River Delta were selected first, based on both economic and geographical representation and the proportion of migrants [14]. Then, based on the final sample size requirement (see next section for details), we randomly selected factories in shoe making, electronic, and plastics industries from each city by applying computer-generated random numbers to the city’s list of factories. The county CDC (Occupational Health Institute) assisted in the selection process and helped with the selection of local enterprises to participate in the study. Finally, workers were randomly selected from the sampled factories for participation.

### Sample size determination

Guided by the sample size estimation method, n=Za22P^1-P^Nδ2N-1+Za22P^1-P^, where P^ was the estimated rate of poor and moderate work ability (50%); N was the Pearl River Delta’s total population based on the 2010 census (approximately 60 million); $\delta =0.05,\;Z_{\frac{a}{2}}=1.96,$ we determined that a minimum sample of 385 was needed. After considering three additional factors such as estimated response rate (80%) and multi-variable and multi-model analysis (normally 1.7 times the univariable and single-model analysis), we further increased the sample size to 818 (385 × 1.7/0.8 = 818). The total sample was then assigned to the four selected cities proportional to the cities’ population distribution (47%, 26%, 15%, and 12%, for Guangzhou, Foshan, Zhaoqing, Qingyuan, respectively).

### Research participants

In this cross-sectional survey, a total of 1,000 migrant workers from ten factories in Guangzhou, Foshan, Qingyuan, Zhaoqing of the Pearl River Delta were randomly selected to answer the study questionnaire. During the survey which took place between September and October 2012, after the investigators explained the purpose of the study and the contents of the questionnaire, migrant workers then completed the questionnaire by themselves. 907 of them completed the questionnaire with a response rate of 90.7%.

Eligible participants were required to have met the following criteria: 1) They were to have non-local “Hukou”; 2) They were to be first-line production workers working in manufactory plants; 3) They were to have left their “Hukou” registered hometown for at least 3 months; and 4) They were willing to provide verbal informed consent. Exclusion criteria included: 1) team leaders or management personnel; or 2) having difficulty communicating, such as reading or answering the study questionnaire.

### Ethics statement

The study was approved by the Institutional Review Board (IRB) of the School of Public Health at Sun Yat-sen University. Written informed consent was obtained from all study participants.

### Measurements

#### Work ability

The work ability index (WAI) was determined on the basis of the answers to a series of questions which took into consideration the demands of work, the worker’s health status and resources. Each worker completed the questionnaire in the presence of an occupational health professional who rated the responses according to the instructions. WAI is a summary measure of seven items (ranging 7–49) [11]:

1) Current work ability compared with the lifetime maximum (0–10);

2) Work ability in relation to the demands of the job (2–10);

3) Number of current diseases, diagnosed by a physician (1–7);

4) Estimated work impairment due to diseases (1–6);

5) Sick leave during the past year (12 months) (1–5);

6) Own prognosis of work ability 2 years from now (1–7);

7) Mental resources (1–4).

To analyze the impact of independent variables on different domains of WAI, we combined 7 items of WAI into 3 domains according to the purpose of WAI: 1) perceived work ability, including items 1, 2 and 6 of WAI; 2) worker’s health status, including items 3, 4 and 5 of WAI; and 3) mental resources, including item 7.

There were three reasons which we divided WAI into three domains: 1) The WAI was determined on the basis of the answers to a series of questions which took into consideration the demands of work, as well as the worker’s health status and resources [11]. 2) Other research has used these classifications [15]. 3) We also carried out confirmatory factor analysis through principal component analysis, selecting factors with eigenvalues greater than 1 and correlation coefficients greater than 0.50. The results were the same as in previous research [15].

#### Migration characteristics

Migration characteristics were mainly focused independent variables in this study. Migration characteristics include length of migration, permanent migration intention and social support. Length of migration was based on the self-reported calendar year in which the participants first left their hometown to search for a job in another city. Permanent migration intention was measured by an item asking participants whether they wanted to have an urban “hukou” and reside in urban cities, and the response categories were yes and no.

Social support was measured by the Chinese Social Support Assessment Scale which has been widely used in China [16]. It has 3 dimensions: named objective support, subjective support, and accessibility of support, and the scores ranged from 5 to 44. The score from the scale was used as a continuous variable.

#### Individual characteristics

Individual characteristics were used as control variables in this study. In previous research, age, gender, marital status, income, and education have been the most commonly researched individual factors. Since most of our subjects’ income ranges were narrow (approximately 2,500 RMB per month), and most of them had below high school educational levels, we did not use income and education as control variables. Rather,, age, gender, and marital status were collected and used as control variables.

#### Work-related factors

Work-related factors were used as control variables in this study. A large variety of work-related factors have been addressed in previous cross-sectional studies [13]. These factors can be divided into two categories: 1) Work-related physical factors, such as thermal discomfort [17], physical climate, muscular work, and restless work environment [18]. 2) Work-related psychosocial factors, such as poor management and lack of freedom [18]. The work-related factors in this study consisted of items related to the physical work environment and psychosocial work-related factors.

Physical work environment was measured by two five-point Likert type scales asking, “Are you exposed to disturbing or tiresome noise at work?” and “Are you exposed to excessive heat, cold or draught at work?” Response categories were “nearly all of the work day, roughly ¾ of the work day, half of the work day, roughly ¼ of the work day, and rarely (below 1/10 of the time)”, which were coded as 1, 2, 3, 4, and 5. The sum of the two items was used as a continuous variable.

Psychosocial work-related factors include work control and work pressure. Work control was measured by two five-point Likert-type scales asking the participants whether “I can express my opinion about things I care in my workplace”, and whether “I am involved in decision-making about things related to me or others at my workplace”. Response categories were “strongly agree, agree, neutral, disagree, and strongly disagree”, which were coded as 5, 4, 3, 2, and 1. The sum of the two items was used as a continuous variable.

Work pressure was measured by a two five-point Likert-type scale asking the participants if they agree with the following statements, “I feel stressed at work”, and “I feel excessively stressed during my work”. Response categories were the same as those in work control. The sum of the two items was used as a continuous variable.

### Statistical analysis

A database was constructed using Epidata 3.0 and statistical analyses were performed using SPSS version 20.0. First, as part of a test of reliability, internal consistency was examined using Cronbach’s alpha. Based on the criterion that a satisfactory alpha coefficient should be equal to or greater than 0.60, the measurements were deemed reliable. Specifically, the scales used to measure the three domains of WAI, physical environment, work control, work pressure, and social support, demonstrated a reliability alpha coefficient of 0.65, 0.64, 0.64, 0.81, 0.82, 0.84, and 0.76, respective, suggesting that all of the dimensions had an acceptable reliability coefficient.

All descriptive data are presented as means and standard deviations, or percentages when appropriate. The participants’ WAI scores were classified into the following four categories: low, moderate, good, and excellent work ability. In categorizing, we used three cutoff points (the 15th percentile, the median, and the 85th percentile), based on the current distribution of the scores for young employees [19]. This procedure resulted in16% of workers being classified into the poor work ability category (index scores 15–35), 30.9% into the moderate category (index scores 36–40), 40.7% into the good category (index scores 41–45), and 12.5% into the excellent category (index scores 46–49). The sum of the score of each domain were calculated and the medians were used to classify the workers into the lower score or higher score group.

Then, bivariate analysis was conducted to examine the relationship between individual, work related factors and migration characteristics, and WAI grade, using *X*^2^ for categorical measures and ANOVA for continuous measures. Statistical significance was set at P ≤ 0.05.

Finally, logistic regressions were applied to assess the influences of the independent variables on the dependent variables. The dependent variables are WAI grade, and levels of three domains of WAI. Due to the hierarchical sampling process used in this study, a multilevel modeling strategy was used to analyze the data. A multilevel logistic model was used to assess the influencing factors on each domain of WAI. According to the principle of applying a multilevel Model, ICC (Intra-class Correlation Coefficient) should be calculated first to identify whether between-group heterogeneity existed among the different factory groups. If ICC is significant, multi-level model should be adopted. Otherwise, a general multiple regression model could be used. First we calculated the ICC and the result showed that the ICC was lower than 3%, which means that it is not necessary to use multilevel model. Therefore we used multiple logistic models to assess influencing factors on WAI.

## Results

Table 1 shows the description of individual, work-related and migration characteristics, and WAI grade of the study population. The mean work ability index was 40.1 (SD 4.6), ranging from 17 to 49. The distribution of excellent, good, moderate and poor work ability was 12.5%, 40.7%, 30.9%, and 16.0%, respectively. The mean age of the workers was 30.3 years, ranging from 16 to 56 years. 44.5% were females and 54.5 males. The mean length of migration was 7.8 years (SD 6.2). The distributions of age, gender and length of migration were almost the same as the characteristics of migrant population in Guangdong Province. However, most of the workers were between the ages of 20 and 45 years, and those above 45 years were scarce. The mean score of evaluation on physical environment was 6.6 (with 10 being the best score), which shows that the working environment was not good. Perceived work control and work pressure were 6.8 and 6.2 (with 10 indicating greatest work control and work pressure). 38.7% of workers reported permanent migration intention. However the remainder wanted to go back to their hometown in the future.

**Table 1:** Description of individual, work-related and migration characteristics, and WAI grade of migrant workers in PRD of China

	**Mean**	**SD**	**N**	**%**
Age	30.3	9.1	907	
Gender				
Female			404	44.5
Male			503	55.5
Marital status				
Single			378	41.7
Married			529	58.3
Physical environment (2–10)	6.6	2.4	907	
Work control (2–10)	6.8	1.7	907	
Work pressure (2–10)	6.2	1.9	907	
Length of migration (year)	7.8	6.2	907	
Permanent migration intention				
Yes			351	38.7
No			556	61.3
Social Support (5–44)	22	5.1	907	
WAI (17–49)				
Poor ≤ 35			145	16.0
Moderate (36–40)			280	30.9
Good (41–45)			369	40.7
Excellent (>45)			113	12.5
Y1 = Perceived work ability				
Weak			497	54.8
Strong			410	45.2
Y2 = Health status				
Bad			615	67.8
Good			292	32.3
Y3 = Mental resource				
Weak			409	45.1
Strong			498	54.9

Table 2 describes the bivariate analysis between individual, work related factors and migration characteristics, and WAI grade based on for categorical measures and ANOVA for continuous measures. Among migration characteristics, permanent migration intention was not significantly associated with WAI. However, longer length of migration and poor social support were significantly associated with poor WAI. Age and marital status were associated with WAI grade. Lower reporting of impact by noise, heat, cold or draught, like higher perceived work control and lower work pressure, were significantly associated with better WAI.

**Table 2:** Relationship between individual, work-related and migration characteristics and WAI grade of migrant workers in PRD of China

	**Poor**	**Moderate**	**Good**	**Excellent**	**F/*χ***^**2**^	**Sig.**
Age	27.7±7.7	29.3±9.1	30.7±9.3	35.0±8.4	15.91	***
Gender						
Female	60 (14.9)	121 (30.0)	176 (43.6)	47 (11.6)	25.85	***
Male	85 (16.9)	159 (31.6)	193 (38.4)	66 (13.1)		
Marital Status						
Single	72 (19.0)	133 (35.2)	148 (39.2)	25 (6.6)	2.67	0.445
Married	73 (13.8)	147 (27.8)	221 (41.8)	88 (16.6)		
Physical Environment (2–10)	5.8±2.4	6.4±2.2	6.8±2.3	7.6±2.3	14.67	***
Work Control (2–10)	6.1±1.6	6.8±1.6	6.9±1.7	7.5±1.7	16.86	***
Work Pressure (2–10)	7.0±1.9	6.4±1.8	6.1±1.9	5.2±1.9	21.84	***
Length of Migration (year)	7.2±5.7	7.3±6.0	7.8±6.3	9.5±6.4	4.23	**
Permanent Migration Intention						
Yes	53 (15.1)	108 (30.8)	149 (42.5)	41 (11.7)	1.00	0.801
No	92 (16.5)	172 (30.9)	220 (39.6)	72 (12.9)		
Social Support (5–44)	20.2±4.6	20.8±4.8	22.3±5.2	23.2±5.4	12.28	***

***:p<0.001, **:p<0.01, *:p<0.05.

Based on *χ*^2^ for categorical measures and ANOVA for continuous measures.

Table 3 shows the results of logistic regression models. In total, there were four models in our study, in which the dependent variables were WAI grade (using ordinal multinomial logistic model), perceived work ability, health status, and mental resources (using binary logistic regression), respectively. Age, work pressure and social support all had significant association with the dependent variables in these models. However, gender, marital status, physical environment, work control, length of migration and permanent migration intention played different roles in them.

**Table 3:** Logistic regression of individual, work-related, and migration characteristics with work ability index among migrant workers in PRD of China

	**Y = WAI**	**Y1 = Perception of work ability**	**Y2 = Health Status**	**Y3 = Mental resource**
	**OR (95% CI)**	**OR (95% CI)**	**OR (95% CI)**	**OR (95% CI)**
Age	1.04 (1.02,1.07)***	1.03 (1.01,1.05)*	1.03 (1.01,1.06)**	1.04 (1.02,1.07)**
Gender (Female #)	1.20 (0.93,1.56)	1.08 (0.80,1.45)	1.08 (0.79,1.47)	0.91 (0.67,1.23)
Marital status (Not married #)	0.98 (0.70,1.39)	1.25 (0.85,1.85)	0.81 (0.53,1.22)	1.13 (0.76,1.70)
Physical environment/2-10	1.05 (1.05,1.17)***	1.11 (1.04,1.18)**	1.04 (0.97,1.11)	1.14 (1.07,1.22)***
Work control/2-10	1.19 (1.11,1.28)***	1.17 (1.08,1.28)***	1.08 (0.99,1.18)	1.17 (1.07,1.28)***
Work pressure/2-10	0.82 (0.77,0.88)***	0.92 (0.86,0.99)*	0.78 (0.73,0.85)***	0.82 (0.76,0.89)***
Length of migration/Year	0.99 (0.97,1.01)	1.02 (0.99,1.05)	0.98 (0.95,1.01)	0.97 (0.94,0.99)*
Permanent migration intention (No #)	1.10 (0.86,1.42)	1.24 (0.93,1.65)	1.15 (0.85,1.56)	0.65 (0.48,0.87)**
Social support/5-44	1.05 (1.02,1.07)***	1.04 (1.01,1.07)**	1.03 (1.01,1.06)*	1.07 (1.03,1.10)***

***:p < 0.001, **:p < 0.01, *:p < 0.05.

#Reference group.

Model 1 reveals that among migration characteristics, better social support had a positive and significant relationship with work ability. In addition, younger migrant workers those and who reported worse physical environments, worse work control and higher work pressure have lower work ability. In model 2, the dependent variable was perceived work ability. Worse social support, younger age, worse physical environment and work control and high work pressure were all significantly related to weak perceived work ability. In model 3, older age and less work pressure were found to have a significant relation to good health status.

Many factors were associated with mental resource. Older age, better physical environment, better work control, less work pressure, permanent migration intention and better social support were all significantly associated with better WAI.

## Discussion

The main purpose of this study has been to explore the relationship between migration characteristics and work ability. The results show that migration characteristics, especially social support are significantly associated with the work ability index of migrant workers in the Pearl River Delta. WAI was also influenced by individual and work-related factors.

Few studies have focused on the effects of social support on work ability. A study of cancer survivors in Sweden reported that support from supervisors and co-workers were significantly associated with work ability [20], which was similar to the findings of our study. Besides the relationship of social support and work ability, we also found an association between social support and health status/mental resource dimensions of work ability. No other studies have analyzed the factors associated with the health status/mental resource dimensions of work ability, but substantial evidence has accumulated over the past few decades showing that social support is positively and causally related to mental health and physical health [21-28]. A study of migrant workers in Shanghai also suggested that social support acted as a moderator to decrease the stress of migration and to improve migrants’ mental health [9].

**Figure 1:**
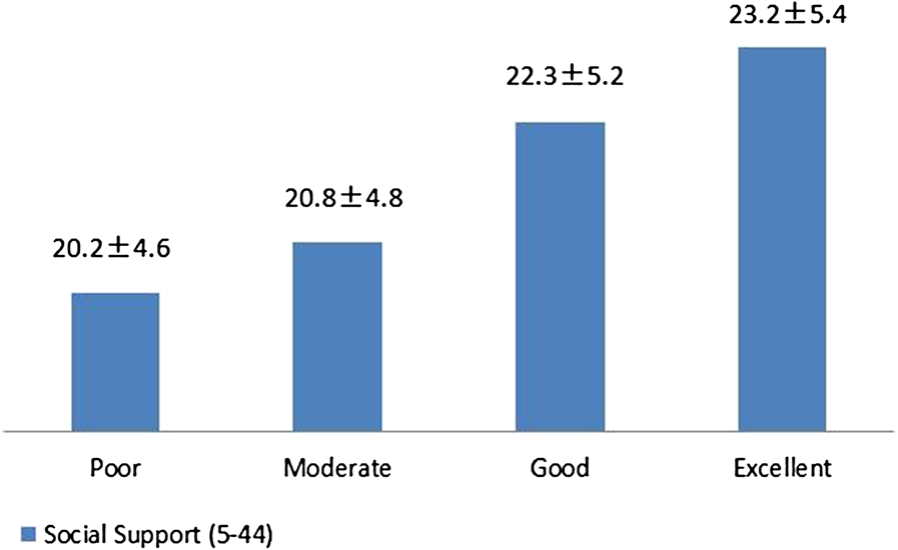
Social support scores among four grade of WAI.

**Figure 2:**
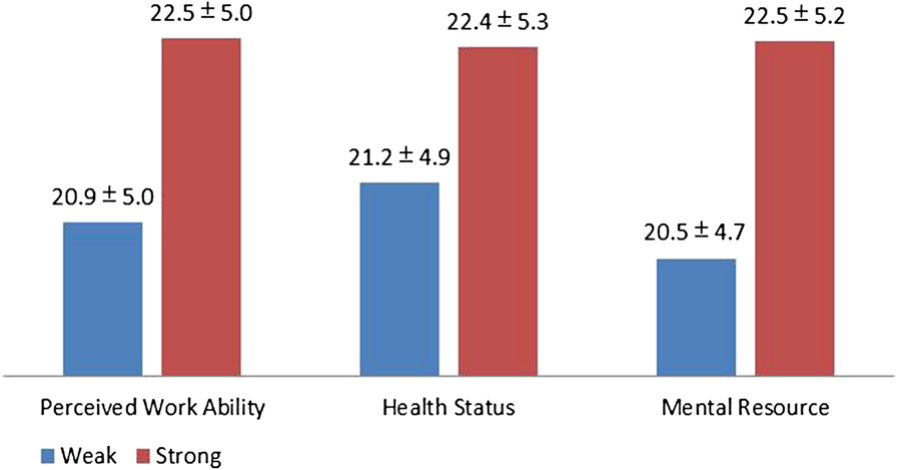
Social support scores among weak and strong groups of three domains of WAI.

Social support comes from spouses, children, family members, colleagues, and friends. In China, most people migrate individually, or only with several close family members, since the living cost in urban cities is high. Thus the support from the family may be weak. But it is well-known in China that numerous migrant communities (or migrant village) have been established in big cities, some of which have been developed, demolished and eventually reconstruction [10,29]. Most of these migrant communities have been organized around migrants’ common place of origin, while some have been organized around urban occupation (e.g. factory workers, construction workers), leading to increased camaraderie and mutual support [10]. Results from a study in Hangzhou show that 71.5% of migrants seek help from friends and colleagues when they need help [7]. This result reveals the importance of social support on work ability, which makes it possible to improve migrant workers’ work ability by promoting social support, not only from family members, but also via other sources, such as labor union and local communities (Figures 1 and 2).

Permanent migration intention was found to be associated with the mental resources domain of WAI. A study in the Pearl River Delta had similar results. The result of that study revealed that migrant workers who are more eager to pursue urban employment and to identify with urban life, but have less favorable work and life experience in the city, tend to permanently migrate to urban areas [30]. The differences between their expectancy and the reality of urban life may lead to more psychological pressure, which makes migrant workers more eager to be accepted by their urban environs. However, in China, migrant workers encounter unequal treatment on the job and in the urban environment, ranging from employment discrimination and lack of social security benefits to lack of access to compulsory education and public goods. Allowing migrant-workers to obtain urban household registration so that they can remain permanently in the cities where they work is seen as a fundamental solution to the present inequities and the social problems resulting from this large-scale rural–urban labor circulation.

Length of migration has a significant negative association with the mental resource score of WAI. This is similar to the “Healthy Migrant Phenomenon”. In the initial phase of migration, there may be fewer specific health problems because of a favorable age structure, prior medical screening in some cases and a shorter duration of stay. However, the longer the stay, the family may arrive to join the primary migrant and may produce different levels and types of stress. O’Degaard [31] had reported that the rates of schizophrenia among Norwegians in a US sample were higher among those who had been in the country for 10–12 years. But another study in Beijing found that length of migration was not significantly associated with the mental health of migrant workers [32]. This reminds us that it is likely that the immediate impact of migration will not necessarily lead to mental problems, but when the migrant begins to settle down they will be affected by social and economic factors which lead to mental stress. This implies that migrant workers who live in an urban are for a longer duration would pay more attention to their mental problems.

In addition to migration characteristics, the relationship between work ability and individual factors and work-related factors was also observed in our study. Age was a significant factor associated with WAI in many studies, but the direction of the association was inconsistent. Some studies reported a decreased WAI with older age [33-35,17]. Other studies showed no association [36], and some found a higher risk for poor WAI among younger workers [37]. In our study, older migrant workers had higher WAI than younger workers. The population of our study consisted mainly of young migrant workers (mean age 30.3). Hence, the negative association is most likely due to a strong “healthy worker selection effect”.

Work-related physical factors influenced not only the health related dimension of WAI, but also the mental resource dimension. A poor physical environment was associated with poor WAI. This was also demonstrated in other studies [13,17]. Besides work-related physical factors, work control and work pressure were also found to have a significant association with the work ability and mental resources dimension of WAI. Similar results have been reported in other studies [36]. These findings suggest that improving migrant workers’ physical working condition, increasing their work control and decreasing their work pressure may promote migrant workers’ work ability and health.

Several limitations of this study must be taken into account. First, the cross-sectional nature of the study did not permit further exploration of causal relationships between these factors and work ability. Second, the data were drawn from workers on duty, and workers taking sick leave were not included in our study. Therefore, selective participation could have affected the generalizability of the study as well as have influenced the results. Finally, the variability explained by migration characteristics was low, showing that migration characteristics may not be an important factor influencing WAI.

## Conclusions

This study deepens the understanding of factors influencing work ability from the perspective of migration. We found that among migration characteristics, social support was positively associated with work ability. Permanent migration intention and a longer length of migration were negatively associated with the mental resource domain of WAI. Better physical working environments, better work control and lower work pressure also has a positive relationship with work ability. Our primary policy suggestion was to promote migrant workers’ social support in order to improve their work ability. Migrant workers with a longer length of migration and permanent migration intention need to have more attention focused on their mental problems. Improving migrant workers’ physical and psychosocial related work environments would also increase their work ability.

## Competing interests

The authors declare that they have no competing interests.

## Authors’ contributions

LH designed the study, the migrant worker survey, and the statistical analysis and drafted the manuscript. LS oversaw the study design and participated in drafting the manuscript. LLu participated in the survey of migrant workers and data analysis. LLi participated in the design of the study and was the study’s PI. All authors read and approved the final manuscript.

## Pre-publication history

The pre-publication history for this paper can be accessed here:

http://www.biomedcentral.com/1471-2458/14/353/prepub
